# Pain perception in schizophrenia: A neglected phenomenon with a great impact

**DOI:** 10.1192/j.eurpsy.2021.1165

**Published:** 2021-08-13

**Authors:** N. Faouel, B. Ben Mohamed, M. Bejar, F. Zaafrane, L. Gaha

**Affiliations:** 1 Research Laboratory, R05es10, Faculty Of Medicine Of Monastir, Tunisia, Psychiatry Department, Fattouma Bourguiba University Hospital, Monastir, Tunisia, monastir, Tunisia; 2 Psychiatry, Faculty of medicine of Monastir, Monastir, Tunisia

**Keywords:** schizophrénia, pain threshold, nociceptors, Pain

## Abstract

**Introduction:**

A decrease in pain sensitivity has been observed in patients with schizophrenia since the beginning of the twentieth century. This hypothesis further emerged during the last decades due to many clinical findings.

**Objectives:**

To study pain responsiveness in patients with schizophrenia and explore its physiopathological mechanism through a review of the literature.

**Methods:**

We searched the Medline database with no time restrictions, and we hand searched the references of all retrieved reviews. After removing duplicates, we selected Full-text articles in both French and English languages. Keywords: “schizophrenia”, “pain”, “pain threshold”, “nociceptors”, “opioid receptors”, “opioid peptides”

**Results:**

We have collected 399 references, we finally included 50 Articles only. Many case reports with heterogeneous types of pain concluded that despite the high prevalence of somatic comorbidities in patients with schizophrenia, there was no significant difference in pain complaints between patients with schizophrenia and controls. There was a positive correlation between the decrease in pain sensitivity and schizophrenia. Experimental studies supported a decrease in pain perception and a high pain threshold in those patients. The neurobiological hypothesis suggested the lack of pain transmission by the dysfunctional glutamatergic system and the involvement of the opioid system. these findings have been reported in patients even before starting treatment. The psychopathology theory pointed to the impact of psychotic defenses such as denial and cleavage in the phenomenon of pain insensitivity.

**Conclusions:**

The meticulous research of pain symptoms should be systematic in patients with schizophrenia and the hypoalgesia should be considered when dealing with somatic conditions in this specific population.

